# Erratum: Phylogenetic analysis of metastatic progression in breast cancer using somatic mutations and copy number aberrations

**DOI:** 10.1038/ncomms15759

**Published:** 2017-06-06

**Authors:** David Brown, Dominiek Smeets, Borbála Székely, Denis Larsimont, A. Marcell Szász, Pierre-Yves Adnet, Françoise Rothé, Ghizlane Rouas, Zsófia I Nagy, Zsófia Faragó, Anna-Mária Tőkés, Magdolna Dank, Gyöngyvér Szentmártoni, Nóra Udvarhelyi, Gabriele Zoppoli, Lajos Pusztai, Martine Piccart, Janina Kulka, Diether Lambrechts, Christos Sotiriou, Christine Desmedt

Nature Communications 8: Article number: 14944; DOI: 10.1038/ncomms14944 (2017); Published: 04 21 2017; Updated: 06 06 2017

The HTML version of this Article previously published had an incorrect publication date of 20 April 2017; it should have been 21 April 2017. This has now been corrected in the HTML; the PDF version of the paper was correct from the time of publication.

