# Supplementary material for: Phylogenetic analysis of metastatic progression in breast cancer using somatic mutations and copy number aberrations
Source: Nat Commun. 2017 Apr 20;8:14944. doi: 10.1038/ncomms14944 (PMC5474888; doi:10.1038/ncomms14944)
Supplement: Supplementary Information — Supplementary Figures and Supplementary Notes [file ncomms14944-s1.pdf]

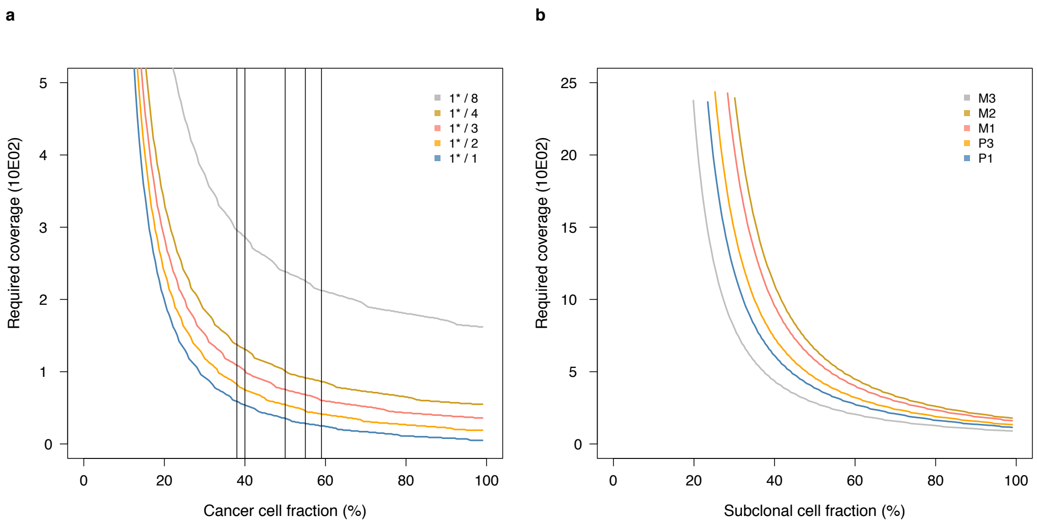

Supplementary Figure 1: Power calculations for samples of patient 8/82. (a) Required depth of sequencing coverage as a function of CCF (%) and CNA in the worst-case scenario of one mutated copy (1*) to reach a statistical power of 95% given a sequencing error rate e = 1.59E-02 and a FPR = 5E-07 to index a fully clonal SNV. The black vertical lines indicate the CCF of the samples as inferred from the respective SNP arrays. (b) Same as in (a) as a function of subclonal cell fraction (%).

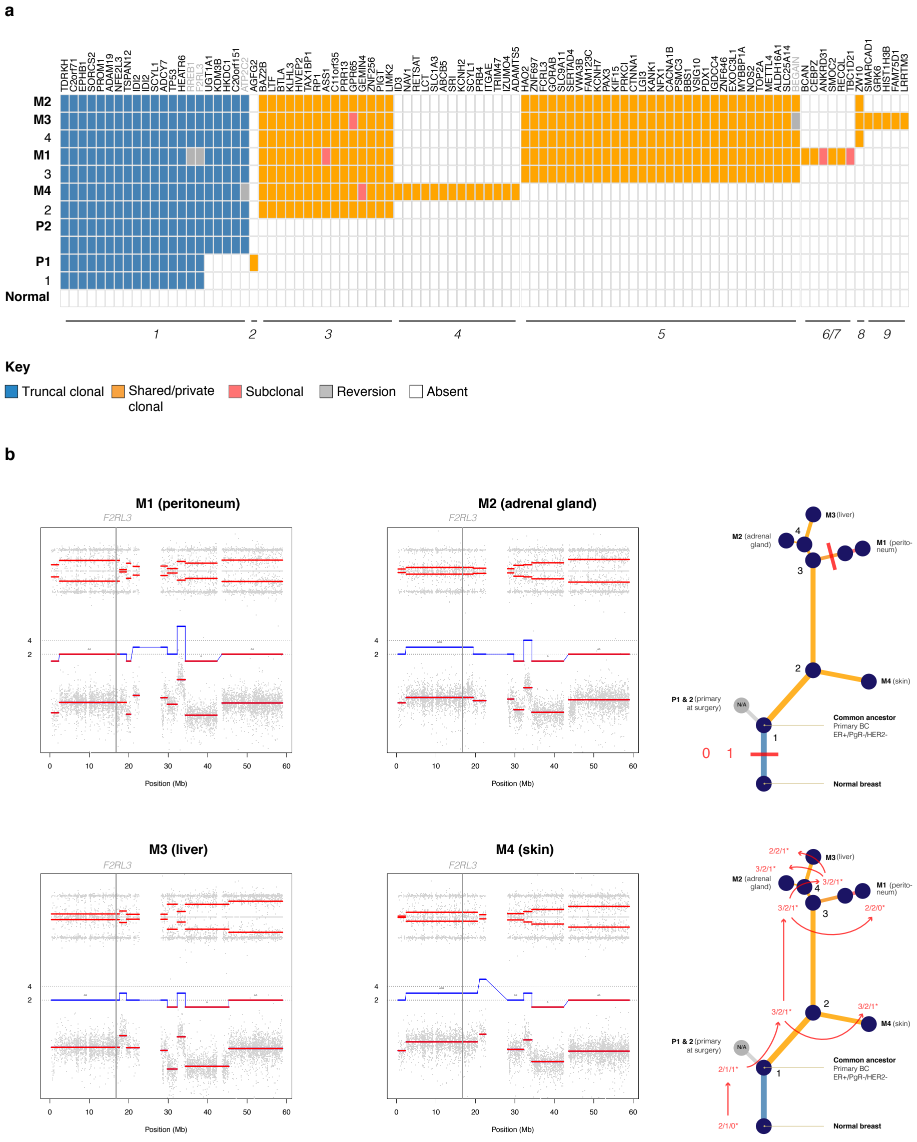

Supplementary Figure 2: Reversions in SNVs are explained by underlying CNAs. (a) Ancestral state reconstruction of tier-3 SNVs for patient 9/68 where 'early' mutations are coloured blue and 'late' mutations are orange. Back mutations are shown in grey. (b) CNA profiles of chromosome 19 for the corresponding metastases where the tracks displayed are in descending order, the BAF, CN estimate and the Log_2 ratios. The genomic coordinate of *F2RL3* is indicated by the vertical grey line. The evolution of the character states i.e. SNV and CN are tracked across the inferred phylogenetic tree on the right

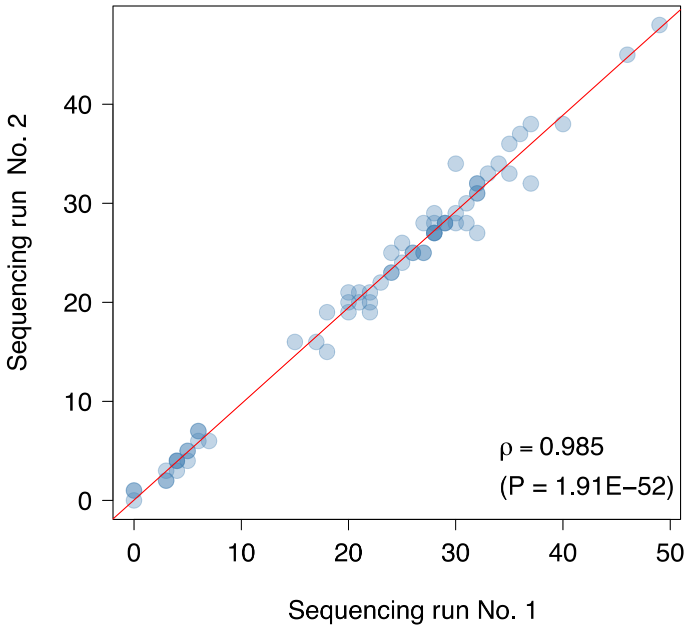

Supplementary Figure 3: Confirmation of the accuracy of VAFs for SNVs. Comparison of the VAFs (%) of SNVs indexed in the metastasis to the adrenal gland of patient 2/57 obtained in two sequencing runs.

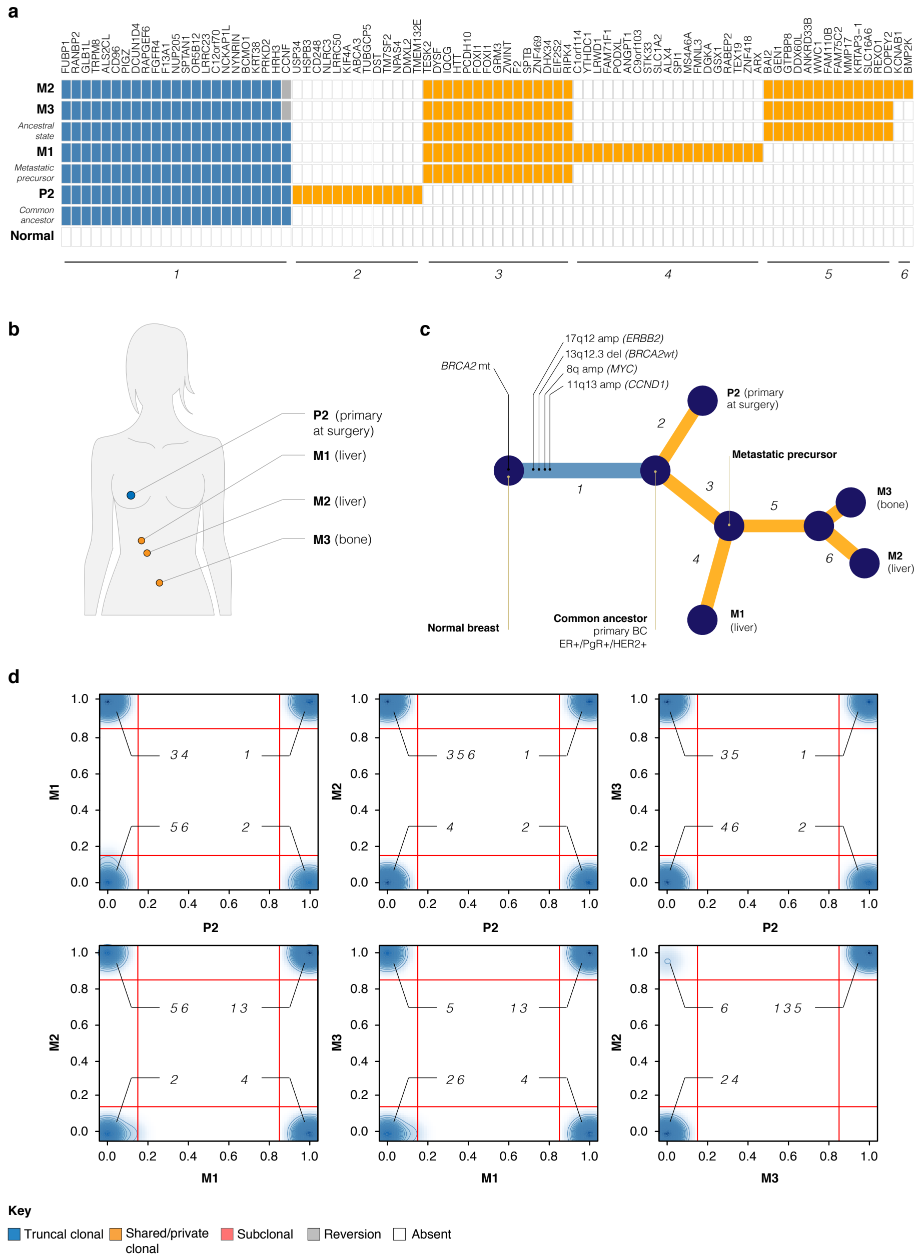

Supplementary Figure 4: Phylogenetic reconstruction of breast cancer progression in patient 5/87. (a) Ancestral state reconstruction of tier-3 SNVs from the primary tumour and three distant lesions of patient 5/87 with anatomic location is shown in (b). (c) Combined phylogenetic tree obtained from CNAs and SNVs and (d) pairwise comparisons of clonal frequencies. The branches of the phylogenetic tree are labelled 1 through 6 in (c) and the location of these mutations in pairwise comparisons is indicated by the corresponding label in (d).

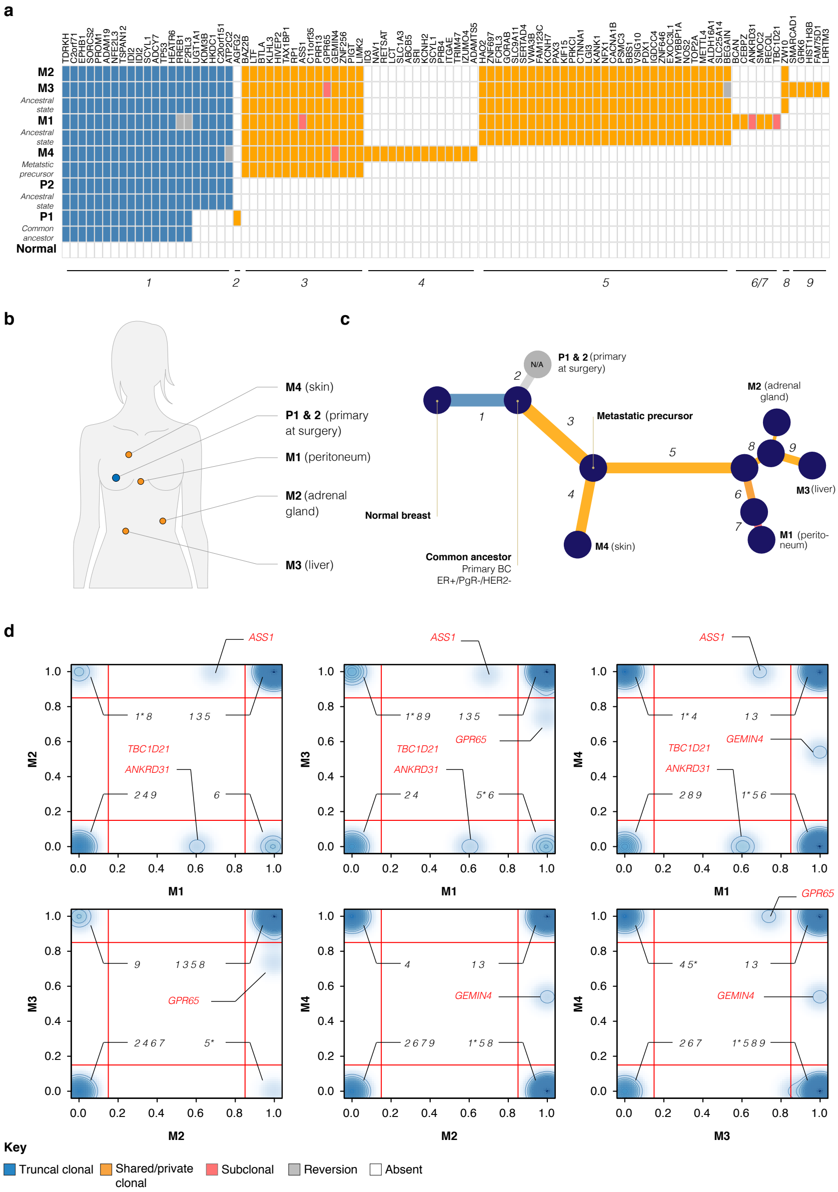

Supplementary Figure 5: Phylogenetic reconstruction of breast cancer progression in patient 9/68. (a) Ancestral state reconstruction of tier-3 SNVs from the primary tumour and four distant lesions of patient 9/68 with anatomic location is shown in (b). (c) Combined phylogenetic tree obtained from CNAs and SNVs and (d) pairwise comparisons of clonal frequencies. The branches of the phylogenetic tree are labelled 1 through 9 in (c) and the location of these mutations in pairwise comparisons is indicated by the corresponding label in (d). 1* and 5* in (d) mark the location of reversions in M1, M3 and M4 that can be explained by CNAs. The clonal structure of three SNVs; ASS1p.M147L, GPR65p.N213fs, and GEMIN4p.C683W, were inconsistent with the phylogenetic reconstruction, being subclonal in M1, M3 and M4 respectively. Considering that ASS1p.M147L and GPR65p.N213fs were fully clonal in the earlier branching M4, imposes that these mutations occurred along branch 1 or 3. Since they were absent from P1 and P2, branch 3 is preferred at the expense of 1. Nonetheless, these represent only 3,2% of tier-4 SNVs and their exclusion does not influence the global topology of the phylogenetic tree. TBC1D21p.Q14K and ANKRD31p.Y1796C are uniquely subclonal in M1 and consistent with the inferred phylogeny.

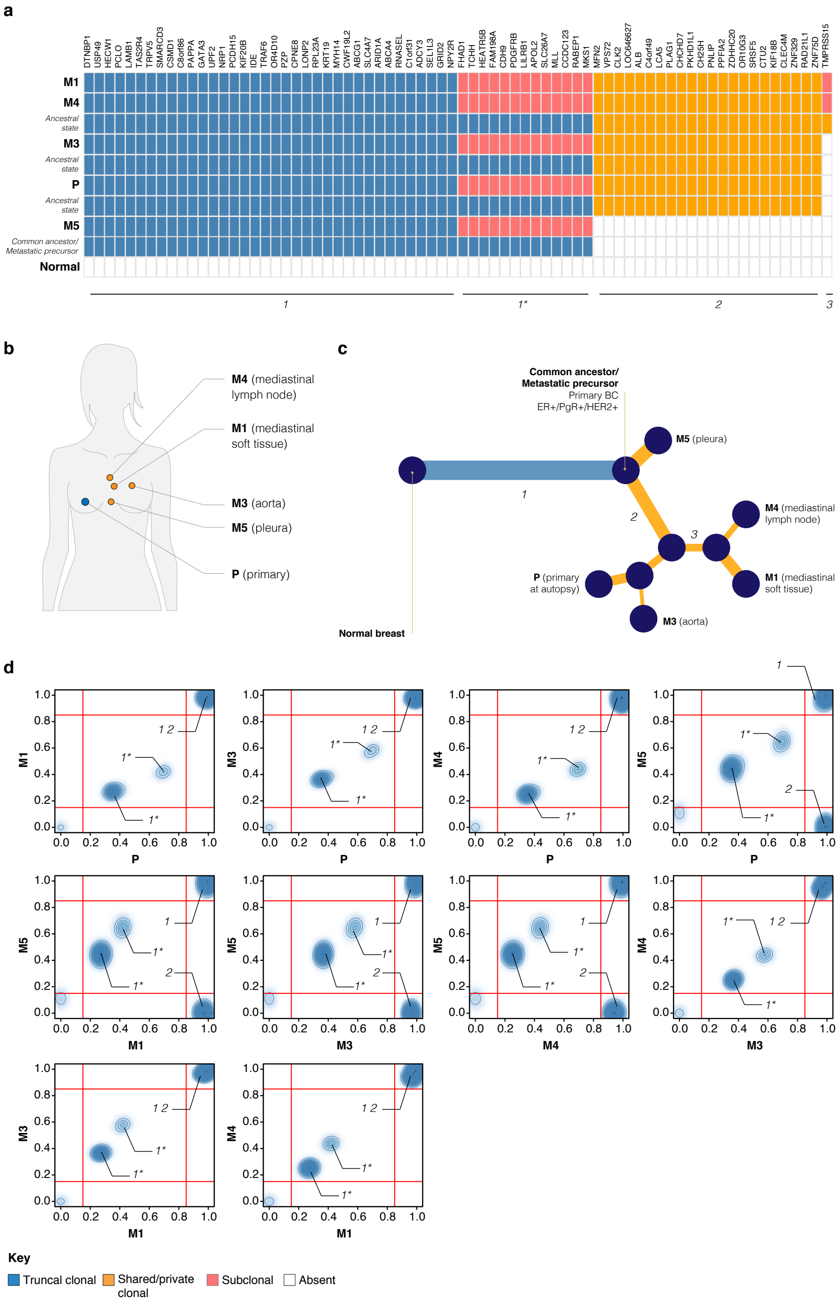

Supplementary Figure 6: Phylogenetic reconstruction of breast cancer progression in patient 1/69. (a) Ancestral state reconstruction of tier-3 SNVs from the primary tumour and four distant lesions of patient 1/69 with anatomic location is shown in (b). (c) Combined phylogenetic tree obtained from CNAs and SNVs and (d) pairwise comparisons of clonal frequencies. The branches of the phylogenetic tree are labelled 1 through 3 in (c) and the location of these mutations in pairwise comparisons is indicated by the corresponding label in (d).

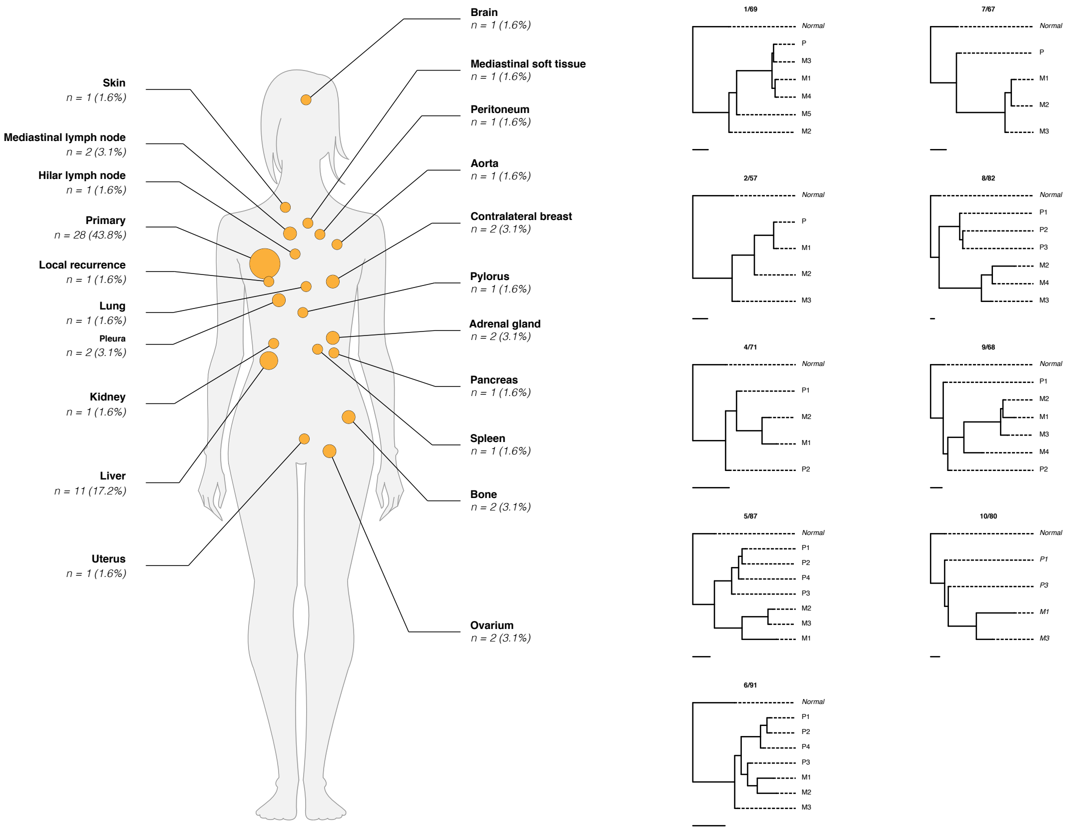

Supplementary Figure 7: Phylogenetic trees obtained from the intermediate-level of tier-3 SNVs using Dollo parsimony. The left most panel provides an anatomic representation of the distribution of tumour deposits whilst the phylogenetic trees inferred from samples with >1500X coverage and >3% VAF are shown on the right. The scale bars at the bottom represent 10 SNVs and give an indication of the total length of the trees.

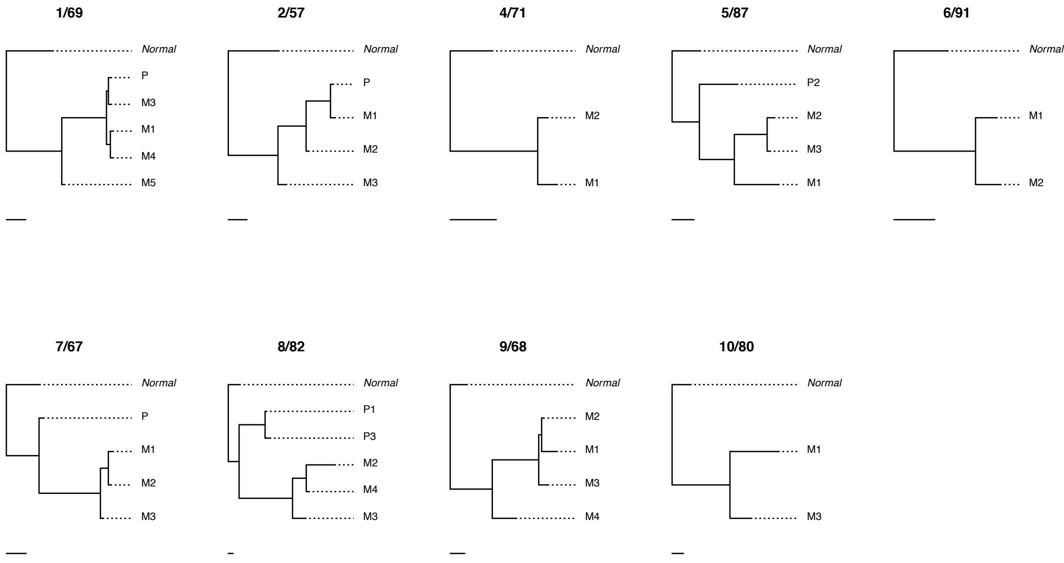

Supplementary Figure 8: Phylogenetic trees obtained from the top-level of tier-4 SNVs using Dollo parsimony. The phylogenetic trees were inferred from samples with >30% CCF, >1500X coverage and >3% VAF. For two patients (4/71 and 6/91), the primary samples did not pass this QC criterion and were not considered further. The scale bars at the bottom represent 10 SNVs and give an indication of the total length of the trees.

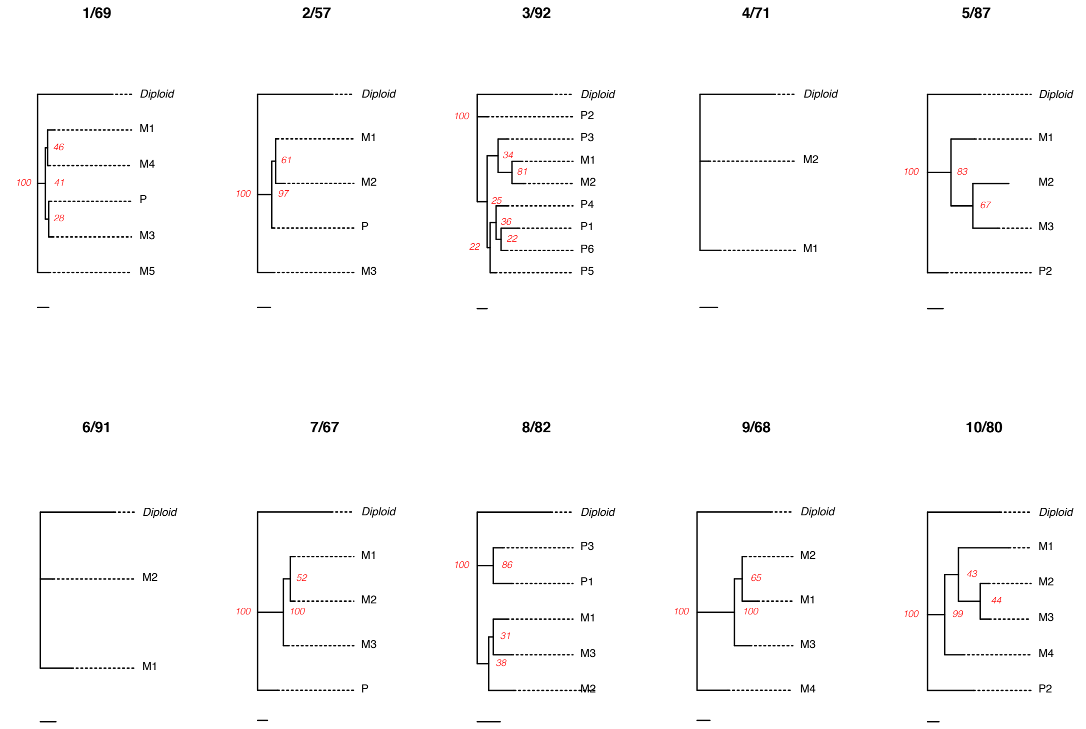

Supplementary Figure 9: Phylogenetic trees obtained from CNAs using MEDICC. The phylogenetic trees were inferred from samples with >30% CCF. For two patients (4/71 and 6/91), the primary samples did not pass this QC criterion and were not considered further. The scale bars at the bottom represent 10 CNAs and provide an indication of the total length of the trees whilst the number besides each node represents the percentage of trees supporting this split in 100 resampling of the distance matrix with added Gaussian noise.

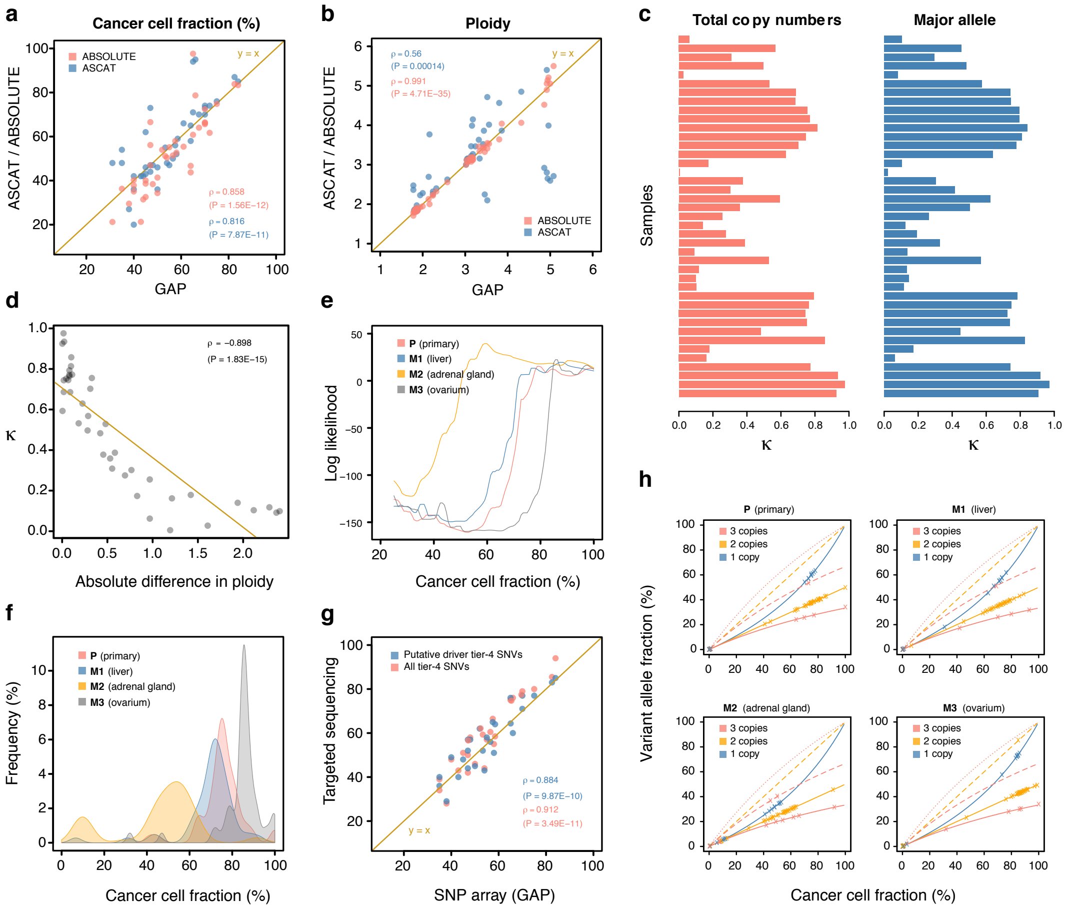

Supplementary Figure 10: Comparison of CCF and ploidy estimates from ASCAT, ABSOLUTE and GAP. (a) Correlation of CCF obtained from ASCAT and ABSOLUTE against the estimate obtained using GAP, (b) same as in (a) for ploidy estimates. In all cases, a high correlation was obtained. However, this high correlation is misleading as there is a large discrepancy in the estimation of total copies and major allele at the level of individual samples. (c) Distribution of κ coefficients obtained by computing a contingency table of either total copies or major alleles for each sample using ASCAT and GAP. In both algorithms, the choice of CCF and ploidy are tightly linked. However, the major source of discrepancy stems from wrong ploidy estimation. This is shown in (d) as the inverse correlation of the κ coefficient with the absolute difference in ploidy estimate between the two algorithms. In extreme cases, we observed differences in ploidy across the matched samples from the same patient. Because GAP allowed for review of ploidy estimates, we opted for this particular algorithm for downstream analyses. Provided the underlying copy number and major allele count are known, the CCF can also be estimated from the VAF of SNVs. (e) Maximization of the Log-likelihood over all tier-4 SNVs of samples from patient 2/57. At the global value of CCF that maximizes this objective function, the CCFs of individual tier-4 SNVs can be represented as a histogram, which provides a rough assessment of clonality. This is shown in (f) and (h) for the same four samples. The correlation of CCF estimated through maximization of the Log-likelihood over all tier-4 SNVs of the CCF of particular tier-4 SNVs with the estimate obtained from the SNP arrays using GAP is shown in (g).

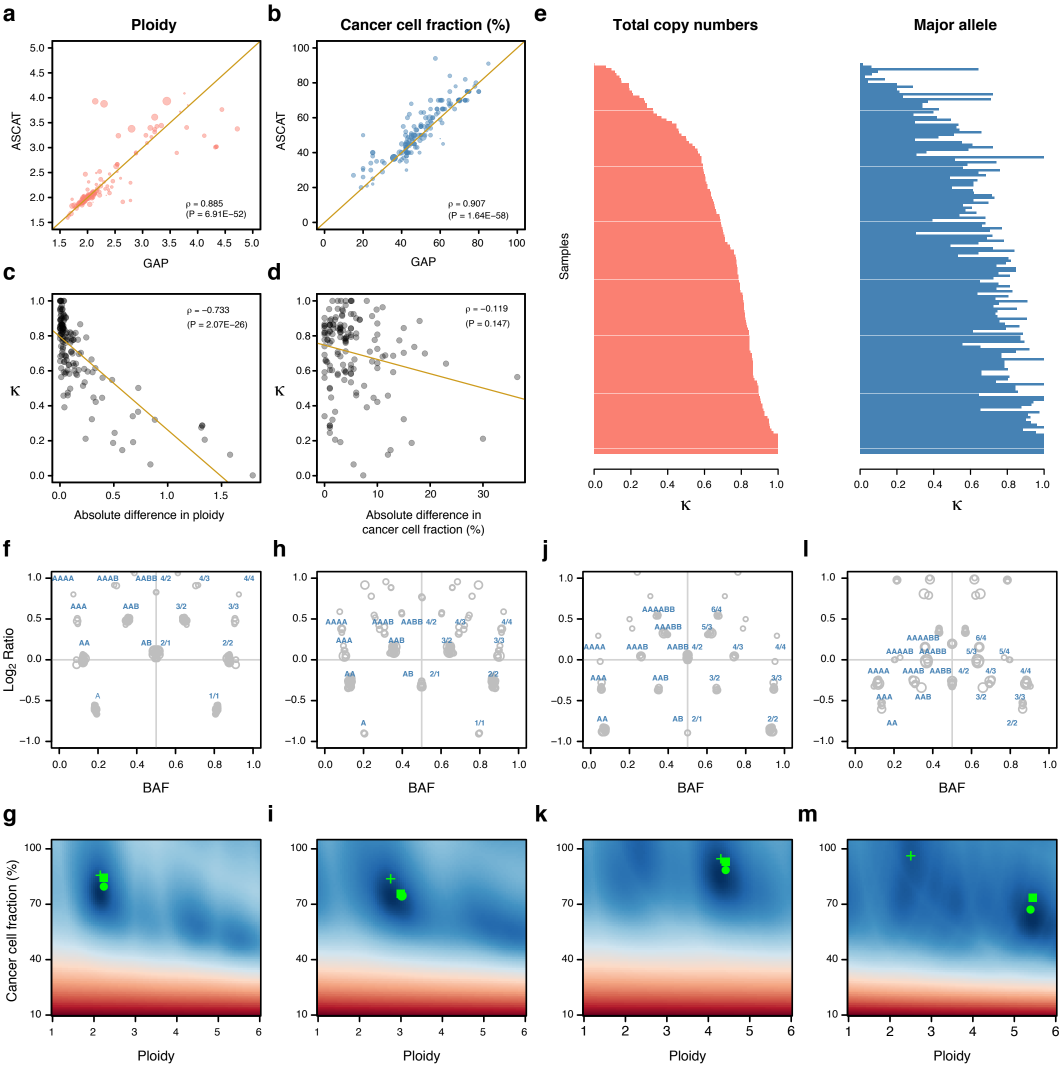

Supplementary Figure 11: Comparison of CCF and ploidy estimate between ASCAT and GAP. Globally, the CCF and ploidy estimates are usually well correlated between ASCAT and GAP. This is shown in (a) and (b). However, this is only apparent and there is often a large variation between the two algorithms at the individual sample level. (e) shows the discrepancy between the two algorithms using the κ coefficient across a series of 125 samples profiled using a similar Affymetrix OncoScan FFPE Express array and subjected to the same QC criteria. (c) and (d) show that this stems mostly from a difference of ploidy estimate rather than CCF as the coefficients were more highly anti-correlated to the absolute difference in ploidy than to the absolute difference in CCF. (f) to (m) show the matched scatter plots of Log_2 Ratio versus BAF (top) and heat maps of CCF versus ploidy (bottom) across a series of samples with increasing genomic mass as an illustration of this problem. + shows to the estimate of ASCAT, $\bullet$ that of GAP and $\blacksquare$ indicates the value obtained from ABSOLUTE.

Supplementary Note 1

Patient 1/69 case report: The patient detected a mass in her breast months before being hospitalized due to anaemia at the age of 78 years. During physical examination, a stage T4 right-sided breast tumour was detected. The patient declined any kind of treatment. Breast cancer was histologically confirmed at autopsy.

Autopsy findings: The primary breast tumour infiltrated the chest wall, the right side of the neck, mediastinal soft tissue, aorta and the outer surface of the left atrium. Metastases were found in the hilar lymph node, mediastinal lymph node and on the pleura. Samples were collected from the primary tumour, aorta wall, mediastinal soft tissue, distant lymph nodes and pleura. Cause of death: advanced stage breast cancer.

Supplementary Note 2

Patient 2/59 case report: The patient was diagnosed with a 1 cm IDC (nuclear grade II, TN) in the left breast at the age of 38. Staging CT scan showed metastases in the lung, liver, and bones. Due to very poor performance status, the patient was not a candidate for CTX or XRT. She died one month after initial diagnosis. This patient was a *BRCA1* germline mutation carrier.

Autopsy findings: The primary tumour was present in the left breast. Metastases were found in the left axillary lymph nodes, hilar lymph nodes, pleura, lung, liver, left adrenal gland, ovarium and bones. Samples were collected from the breast, liver, ovarium and adrenal gland. Cause of death: fulminant liver failure due to liver metastases.

Supplementary Note 3

Patient 3/92 case report: The patient's grandmother on father's side and mother had breast cancer. At the age of 39, during her first pregnancy, she discovered a lump in the right breast; an initial biopsy was negative. Three months later, a biopsy was repeated and revealed an IDC (grade III, TN, Ki67 90%) of 6.5 cm. PET CT scan showed one metastasis in the liver. The patient received six cycles of preoperative TXT-CBP which, based on imaging, resulted in complete response in the liver and axilla, and partial response in the primary tumour. Mastectomy and axillary dissection was performed and revealed an IDC, grade III, TN, Ki67 1%, 40 mm tumour with 1/13 LNs involved (ypT2ypN1). During XRT, the patient had epigastrial symptoms and ultrasound showed multiple liver metastases. Due to her performance status, she was not a candidate for chemotherapy. The patient died 11 months after initial diagnosis.

Autopsy findings: Metastases were found in the liver, distal lymph nodes and on the pleura (samples from the liver and distal lymph nodes). Cause of death: advanced stage breast cancer.

Supplementary Note 4

Patient 4/71 case report: The patient underwent a lumpectomy and axillary dissection for a left side breast tumour at the age of 54. Histological assessment revealed a 2 cm IDC (grade II, TN, Ki67 5%) with one out of seven lymph nodes involved (pT2pN1). The patient received adjuvant systemic treatment of four cycles of FEC. Six months later, the patient underwent mastectomy for a local recurrence. Histological assessment confirmed a recurrence of grade II, TN IDC with muscle involvement and two positive lymph nodes. The patient received five cycles of gemcitabine + docetaxel. One year after initial diagnosis, PET CT scan showed multiple skeletal and liver metastases. Ibandronic acid was started and RFA of a liver metastasis was performed. The patient died 14 months following initial diagnosis.

Autopsy findings: A local recurrence in the left breast and several metastases in the liver and bone (spine) were found. Samples from the liver and the local recurrence were collected. Cause of death: fulminant liver failure due to liver metastases.

Supplementary Note 5

Patient 5/87 case report: The patient's mother had a *BRCA2* germline breast cancer. This mutation was also identified in the patient. At the age of 29, the patient discovered a lump in the right breast. A core biopsy revealed an IDC (grade III, ER+, PgR+, Ki67 20%) associated with high grade DCIS. At diagnosis, HER2/neu status was negative using IHC. In our study, we evaluated centrally the HER2/neu status using FISH. Staging CT-scan detected metastasis in the liver. The patient received a 1st line chemotherapy consisting of three cycles of FAC, with good tumour response in the breast. Mastectomy and axillary dissection revealed an IDC (grade III, ER+, PgR+) with four positive lymph nodes out of 11 (ypT2pN2a). GNRH analogue and letrozole was initiated. PET CT scan showed disease progression in the liver. A second line of chemotherapy with capecitabine and bevacizumab was administered for four months. Eleven months after the initial diagnosis, a disease progression was documented in the liver including the detection of new infradiaphragmatic lymph nodes and bone lesions. A third line of chemotherapy (eight cycles of paclitaxel carboplatin) was administered; and the PET CT scan showed a total metabolic remission in the liver and the bones. At 20 months after diagnosis, a PET CT scan showed disease progression with bone metastases. Zoledronic acid was switched to ibandronate. The patient declined additional chemotherapy. Endocrine therapy and bisphosphonates were given. The patient died 26 months after initial diagnosis.

Autopsy findings: Metastases were found in the liver, lung, pleura, skin, distant lymph nodes, brain and bone. Samples were taken from the liver, lung, brain and bone. Cause of death: hepatorenal syndrome due to disease progression.

Supplementary Note 6

Patient 6/91 case report: The patient's father had bone sarcoma and died at age 28, grandfathers had lung and gastric cancer, aunt had lung cancer. At the age of 38, the patient detected a tumour in the right breast. A core biopsy revealed an IDC (TN, Ki67 10%) with positive axillary lymph node. The PET CT scan did not show any distant metastases. After two cycles of neoadjuvant FEC, the patient discontinued her treatment. At month 12, she returned to the clinic and had a mastectomy and axillary dissection for an ulcerated breast tumour. The histology demonstrated a 60 mm IDC (grade III, TN) with 16 positive lymph nodes out of 16 (ypT4ypN2a). During surgery, a tumour was detected in the contralateral breast leading to a lumpectomy. Histology revealed the presence of a 13 mm IDC associated with ILC (grade III, ER Q-score 3, PgR Q-score 5, Ki67 25%), with one positive lymph node out of two. A PET CT scan detected bone metastases. Bisphosphonates were started. The patient received seven cycles of taxotere-carboplatin as first line chemotherapy which was switched to capecitabine (six cycles) because of allergic reaction to carboplatin. After new skin and liver lesions were detected, the patient died 26 months after initial diagnosis.

Autopsy findings: Metastases were found on the chest wall, skin, in the liver, brain and bones. Samples were obtained from the brain and liver. Cause of death: brain oedema and herniation due to brain metastases.

Supplementary Note 7

Patient 7/67 case report: The patient's mother had breast cancer and father had lung cancer. She underwent lumpectomy and axillary dissection for a tumour of the right breast at the age of 54. Histological assessment revealed a 1.8 cm IDC (grade III, TN, Ki67 4%) without lymph node involvement (0/15); stage pT1cpNo. The patient received adjuvant chemotherapy with four cycles of doxorubicin plus cyclophosphamide followed by radiotherapy of the right breast. Eighteen months after the initial diagnosis, the patient had an epileptiform seizure. MRI showed a brain metastasis. After surgery and whole brain radiation, five cycles of paclitaxel and carboplatin were given. Twelve months later, new lesions were detected in the brain. The patient declined chemotherapy and died 44 months after initial diagnosis.

Autopsy findings: Metastases were found in the brain, lung, kidney, ovarium and spleen. Samples were collected from the lung, spleen, ovarium and kidney. Cause of death: brain oedema and herniation due to brain metastases.

Supplementary Note 8

Patient 8/82 case report: The patient underwent mastectomy and axillary dissection for a right breast tumour at the age of 62. Histological assessment revealed a pleiomor-

phic ILC+LCIS (grade III, ER+ (Q-score 7), PgR-, HER2-, Ki67 6%) with five positive lymph nodes out of seven (pT2 pN1a). The patient received adjuvant chemotherapy with six cycles of FEC followed by XRT and endocrine therapy (exemestane for 16 months followed by letrozole for 19 months). Forty-three months after the initial diagnosis, liver metastases were detected. Radio frequency ablation was performed and endocrine treatment was switched back to exemestane. Disease progression in the liver was documented 16 months later. The patient started fulvestrant for three months. At 61 months after diagnosis, paclitaxel and carboplatin were started due to disease progression. After four cycles, CT scan showed progression in the liver and the apparition of new bone metastases. She received 14 cycles of capecitabine. The patient died 76 months after initial diagnosis.

Autopsy findings: Metastases were found in the mediastinal lymph nodes, pleura, lumbar vertebras, right adrenal gland, liver, rectum, uterus, pylorus, peritoneum and retroperitoneum. Samples were collected from the pleura, pylorus, liver and uterus. Cause of death: advanced stage breast cancer.

Supplementary Note 9

Patient 9/68 case report: The patient underwent mastectomy and axillary dissection for a right-side breast tumour at the age of 68. Histological assessment revealed a 3.8 cm IDC (grade III, ER Q-score 3 and PgR Q-score 2, HER2-, Ki67 5%) without lymph node involvement (0 of 20); stage pT2pNo. The patient received adjuvant endocrine therapy with letrozole for five years. Eighty months after initial diagnosis, a metastasis was found in the sternum. Zoledronic acid was started. Six months later, therapy was switched to ibandronic acid and fulvestrant was initiated as the bone scan detected a new lesion in the femur. Palliative RT was performed for the bone lesions. Seven months later, a CT scan showed multiple metastases in the liver and lung. The patient declined CTX and died 109 months after initial diagnosis.

Autopsy findings: Metastases were found in the liver, lung, adrenal glands, bones, skin and on the peritoneum. Samples were collected from the skin, adrenal gland, liver and peritoneum. Cause of death: cardiorespiratory insufficiency (left ventricle dysfunction, two-sided lung oedema and hydrothorax) due to disease progression.

Supplementary Note 10

Patient 10/80 case report: The patient underwent a lumpectomy and axillary dissection for a right-side breast tumour at age 46. Histological assessment revealed a 2.4 cm IDC, grade III, ER Q-score 4, PgR Q-score 7, Ki67 1% with one positive lymph node out of five (pT2pN1). At diagnosis, the HER2/neu status was negative using IHC. In our study, we repeated the HER2/neu staining using IHC and FISH which demonstrated HER2/neu amplification. The patient received adjuvant chemotherapy, six cycles of

FAC followed by tamoxifen. 64 months after initial diagnosis, a local recurrence was diagnosed. The patient declined surgery and tamoxifen was switched to letrozole. XRT was given. Two years later, metastases were found in the liver. Capecitabine was started (eleven cycles with stable disease). At 111 months, progression was detected in the liver and the breast with new mediastinal lymph nodes metastases. Three cycles of gemcitabine + docetaxel was given which resulted in regression in the breast but progression was detected in the liver and new bone metastases appeared. Mastectomy was performed 115 months after diagnosis (histology: low differentiated IDC). Ibandronic acid was started. Patient received four cycles of gemcitabine + docetaxel. At 119 months, a local recurrence was detected on the right chest wall and RT was given. After finding a new lesion in the contralateral breast, the patient received one cycle of carboplatin. The patient died 123 months after initial diagnosis.

Autopsy findings: Metastases were found in the liver, vertebras, mediastinal LNs and local recurrence on the right chest wall, contralateral breast tumour. Samples were collected from the contralateral breast tumour, mediastinal lymph node, liver and bone. Cause of death: liver insufficiency.

CT: Computed Tomography **CTX:** Chemotherapy **DCIS:** Ductal Carcinoma in situ **FAC:** Fluorouracil + Doxorubicin + Cyclophosphamide **FEC:** Fluorouracil + Epirubicin + Cyclophosphamide **FISH:** Fluorescence in situ hybridization **IDC:** Invasive Ductal Carcinoma **IHC:** Immunohistochemistry **ILC:** Invasive Lobular Carcinoma **LCIS:** Lobular Carcinoma in situ **PET:** Positron Emission Tomography **RFA:** Radiofrequency Ablation **TN:** Triple Negative **XRT:** Radiation Therapy
